# In vivo characterization of white matter pathology in premanifest huntington's disease

**DOI:** 10.1002/ana.25309

**Published:** 2018-09-15

**Authors:** Jiaying Zhang, Sarah Gregory, Rachael I. Scahill, Alexandra Durr, David L. Thomas, Stéphane Lehericy, Geraint Rees, Sarah J. Tabrizi, Hui Zhang, the TrackOn‐HD investigators

**Affiliations:** ^1^ Department of Computer Science and Centre for Medical Image Computing University College London London United Kingdom; ^2^ Huntington's Disease Research Centre, Institute of Neurology University College London London United Kingdom; ^3^ Wellcome Trust Centre for Neuroimaging, Institute of Neurology University College London London United Kingdom; ^4^ Department of Neurodegenerative Disease, Institute of Neurology University College London London United Kingdom; ^5^ ICM – Institut du Cerveau et de la Moelle Epinière, INSERM U1127, CNRS UMR7225, Sorbonne Universités – UPMC Université Paris VI UMR_S1127 and APHP, Genetic department Pitié–Salpêtrière University Hospital Paris France; ^6^ Neuroradiological Academic Unit, Department of Brain Repair and Rehabilitation, Institute of Neurology University College London London United Kingdom; ^7^ Leonard Wolfson Experimental Neurology Centre, Institute of Neurology University College London London United Kingdom; ^8^ Neuroimaging Research Center, Brain and Spinal Cord Institute Pierre and Marie Curie University, Inserm UMR1127, CNRS 7225 Paris France

## Abstract

**Objective:**

Huntington's disease (HD) is a monogenic, fully penetrant neurodegenerative disorder, providing an ideal model for understanding brain changes occurring in the years prior to disease onset. Diffusion tensor imaging (DTI) studies show widespread white matter disorganization in the early premanifest stages (pre‐HD). However, although DTI has proved informative, it provides only limited information about underlying changes in tissue properties. Neurite orientation dispersion and density imaging (NODDI) is a novel magnetic resonance imaging (MRI) technique for characterizing axonal pathology more specifically, providing metrics that separately quantify axonal density and axonal organization. Here, we provide the first in vivo characterization of white matter pathology in pre‐HD using NODDI.

**Methods:**

Diffusion‐weighted MRI data that support DTI and NODDI were acquired from 38 pre‐HD and 45 control participants. Using whole‐brain and region‐of‐interest analyses, NODDI metrics were compared between groups and correlated with clinical scores of disease progression. Whole‐brain changes in DTI metrics were also examined.

**Results:**

The pre‐HD group displayed widespread reductions in axonal density compared with control participants; this correlated with measures of clinical disease progression in the body and genu of the corpus callosum. There was also evidence in the pre‐HD group of increased coherence of axonal packing in the white matter surrounding the basal ganglia.

**Interpretation:**

Our findings suggest that reduced axonal density is one of the major factors underlying white matter pathology in pre‐HD, coupled with altered local organization in areas surrounding the basal ganglia. NODDI metrics show promise in providing more specific information about the biological processes underlying HD and neurodegeneration per se. Ann Neurol 2018;84:497–504

Characterizing brain changes that occur in neurodegeneration prior to symptom onset can provide targets for interventions that may delay or completely arrest the progression of disease.^1^ Huntington's disease (HD) is a monogenic, neurodegenerative disorder, characterized by motor, cognitive, and neuropsychiatric disturbance.^2^ As it is both autosomal dominant and fully penetrant, HD gene carriers can be identified many years prior to clinical onset, enabling neurodegenerative brain changes to be tracked from the earliest stages.

Structural magnetic resonance imaging (MRI) studies have shown robust white matter degeneration in premanifest HD (pre‐HD).^3^, ^4^, ^5^ Neuronal loss initially affects the striatum, but this is followed by reductions in white matter surrounding the basal ganglia, which extend to cortical white matter throughout the cortex. To understand further the nature of these macrostructural changes, it is necessary to investigate underlying alterations in the tissue properties of white matter. This is possible in vivo using diffusion tensor imaging (DTI),^6^ which provides an indirect assessment of white matter microstructure. DTI studies in HD have shown widespread abnormalities in white matter tissue microstructure in pre‐HD gene carriers, with evidence of a widespread reduction in fractional anisotropy (FA; a measure of water diffusion in the direction of the main underlying pathway) and mean diffusivity (MD) in a series of white matter pathways and regions including the corpus callosum, motor regions, and frontal tracts, indicating increased white matter disorganization.^7^, ^8^, ^9^, ^10^, ^11^ It has been suggested that these changes represent axonal degeneration. Animal models of HD, for example, show that mutant huntingtin impairs axonal transport.^12^, ^13^ However, this has yet to be directly observed in vivo in humans, and although the changes observed using DTI are consistent with underlying axonal degeneration in HD, they can also be explained by other microstructural alterations, such as increases in water content.

Recent progress in quantitative MRI has introduced new techniques that can be used to achieve more direct in vivo characterization of white matter microstructure including the potential role of axonal pathology. Neurite orientation dispersion and density imaging (NODDI), for example, applies a 3‐compartment tissue model to multishell diffusion‐weighted imaging (DWI) data, which allows the interrogation of both intra‐ and extracellular properties of white matter tissue and in turn enables differentiation of 2 key aspects of axonal pathology: the packing density of axons in white matter (neurite density index [NDI]) and the spatial organization of the axons (orientation dispersion index [ODI]).^14^ The amount of free water present (free water fraction) is also independently quantified. Using this approach, changes in axonal density can be distinguished from those in axonal spatial organization, while removing the potentially confounding effect of free water, which increases in areas of tissue degeneration.^15^

In the current study, we used NODDI to characterize in vivo white matter pathology during the premanifest stage of HD in a cross‐sectional cohort of 38 pre‐HD gene carriers and 45 controls from the multisite Track‐On HD study.^16^ Using whole‐brain analysis, tract‐based spatial statistics (TBSS),^17^ we examined differences in NODDI metrics (NDI and ODI) between controls and pre‐HD gene carriers in addition to standard DTI metrics (FA and MD) plus axial diffusivity (AD) and radial diffusivity (RD). We then correlated clinical markers of disease progression with NODDI metrics extracted from a set of empirically derived white matter regions of interest (ROIs). We predicted that axonal density reduction would be the main feature of white matter pathology and would correlate with clinical markers of disease progression in pre‐HD.

## Subjects and Methods

### *Participants*

Forty pre‐HD gene carriers and 52 controls were recruited at 2 sites (Paris and London) for the final visit of the Track‐On HD study. Pre‐HD participants were required to have a cytosine–adenine–guanine (CAG) repeat length ≥ 40 and a disease burden score^18^ > 250 at recruitment. Cumulative probability to onset (CPO)^19^ and Unified Huntington's Disease Rating Scale (UHDRS) total motor score (TMS)^20^ were also used as clinical measures of disease progression.

Both these measures aim to estimate disease severity, with CPO representing the calculated distance from onset based on age and number of CAG repeats; TMS is an empirical clinical measure of motor function. Initial recruitment into the Track‐On HD study excluded those with manifest disease, age < 18 or > 65 (unless previously in the Track‐On HD study), major psychiatric, neurological, or medical disorders, or a history of severe head injury; for full details see Klöppel et al.^16^ The study was approved by the local ethics committees, and all participants gave written informed consent according to the Declaration of Helsinki.

### *MRI Acquisition*

Multi‐shell DWI data were acquired for NODDI analysis, and single‐shell DWI data were acquired for standard DTI analysis at both sites using 3T MRI scanners (Tim Trio, Siemens Healthcare, Erlangen, Germany). A twice‐refocused spin‐echo echo‐planar imaging sequence was used to minimize the eddy‐current distortion for both acquisitions. The multi‐shell protocol for NODDI consisted of b values of 2,000, 700, and 300s/mm^2^ with 64, 32, and 8 nonlinear diffusion‐encoding directions, respectively; 14 b = 0s/mm^2^ images; voxel size = 2.5 × 2.5 × 2.5mm^3^; repetition time (TR) = 7,000 milliseconds; echo time (TE) = 90.8 milliseconds; 55 slices; acquisition time = 15 minutes. The single‐shell protocol for DTI consisted of a single b value of 1,000s/mm^2^ and 42 nonlinear diffusion‐encoding directions; 7 b = 0s/mm^2^ images; voxel size = 2 × 2 × 2mm^3^; TR = 13,100 milliseconds; TE = 88 milliseconds; 75 slices; acquisition time = 9.5 minutes.

### *MRI Data Analysis*

Both single‐ and multi‐shell data were initially visually inspected for coverage, motion, and/or ghosting artifacts and to verify minimal eddy‐current distortions. Following motion correction,^21^ NODDI metrics including NDI and ODI were estimated from the multi‐shell data using the NODDI MATLAB toolbox (http://nitrc.org/projects/noddi_toolbox), and DTI metrics including FA, MD, RD, and AD were estimated from the single‐shell data using FSL (https://www.fmrib.ox.ac.uk/fsl).^22^ Individual NODDI and DTI metric maps were then spatially normalized to the group‐specific template for subsequent TBSS and ROI analyses using DTI‐TK (http://dti-tk.sf.net),^23^ a state‐of‐the‐art approach that has been successfully applied to studying white matter in other neurodegenerative diseases.^24^, ^25^

Independent 2‐sample *t* tests were performed in FSL to compare whole‐brain NODDI and DTI metrics between pre‐HD and control groups using permutation testing (n = 5,000) and threshold‐free cluster enhancement^26^ to improve cluster inference, corrected at *p* < 0.05. Age, gender, and site were included as covariates.

ROI analysis was then performed for a set of key white matter tracts chosen a priori according to the literature.^7^, ^8^, ^11^, ^27^ The ROIs included the corpus callosum (genu, body, splenium), the internal capsules (anterior and posterior limbs), and the external capsules (bilateral internal/external capsules were analyzed jointly to increase the statistical power). To minimize partial volume effects, ROIs were defined by thinning masks from the ICBM‐81 white matter atlas^28^ by 1mm. Between‐group comparisons of NODDI metrics for the control and pre‐HD groups were first performed using the general linear model, with age, gender, and site as covariates. The results were false discovery rate–corrected at *p* < 0.05. For ROIs in which significant group differences were identified, the extracted NODDI metrics were correlated with clinical scores of disease progression (CPO and UHDRS‐TMS) corrected at *p* < 0.05.

## Results

### *Demographic and Clinical Characteristics*

Demographic and clinical information is summarized in Table 1. The pre‐HD and control groups were matched for gender and participation at each site; the average age of the pre‐HD group was lower than that of the control group.

**Table 1:** Demographic and Clinical Information

Characteristic	Control	Pre‐HD	*p*
n	45	38	N/A
Age, yr, mean ± SD (range)	49.1 ± 10.8 (28–69)	44.3 ± 8.6 (28–70)	0.03
Gender, F/M	27/18	17/21	n.s.
Site, London/Paris	20/25	18/20	n.s.
CAG repeats, mean ± SD (range)	N/A	42.9 ± 1.9 (40–47)	N/A
CPO, mean ± SD (range)	N/A	0.30 ± 0.18 (0.06–0.75)	N/A
TMS, mean ± SD (range)	N/A	6.40 ± 3.85 (0–15)	N/A

CAG = cytosine–adenine–guanine; CPO = cumulative probability to onset; F = female; M = male; n.s. = not significant; N/A = not applicable; Pre‐HD = premanifest Huntington's disease; SD = standard deviation; TMS = total motor score.

### *Whole‐Brain Analysis*

**Figure 1:**
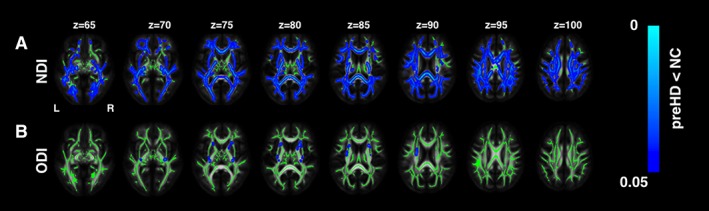
White matter abnormalities: neurite orientation dispersion and density imaging (NODDI) analysis. The regional distribution of differences in NODDI parameters in premanifest Huntington's disease (pre‐HD) gene carriers compared to controls (NC) is shown. There were reductions in neurite density (neurite density index [NDI]) across the whole brain, indicating a reduction in axonal density (A), as well as localized reductions in the dispersion of fibers (orientation dispersion index [ODI]) in the corpus callosum and the internal and external capsule, indicating select pruning of white matter fibers (B), Threshold‐free cluster enhancement (TFCE) *p* < 0.05. Group differences in NODDI metrics are overlaid on white matter skeleton. [Color figure can be viewed at http://wileyonlinelibrary.com]

Whole‐brain analysis of the NODDI data showed widespread reduced NDI in pre‐HD gene carriers when compared with controls (Fig 1A). Reduced NDI was evident in the corpus callosum, the bilateral superior longitudinal fasciculi, the posterior limb of internal capsules, the external capsules, the posterior thalamic radiations, the middle cerebellar peduncles, the corona radiata, the uncinate fasciculi, and the posterior cingulum. ODI was also reduced in more localized regions including the white matter surrounding the basal ganglia, the anterior limb of the internal capsules bilaterally, the external capsules, and the right retrolenticular part of internal capsules (see Fig 1B).

**Figure 2:**
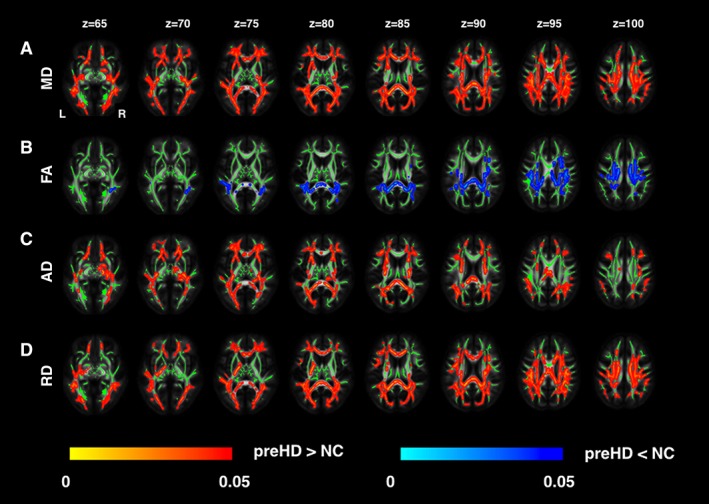
White matter abnormalities: diffusion tensor imaging (DTI) analysis. The regional distribution of differences in DTI metrics in premanifest Huntington's disease (pre‐HD) gene carriers compared to controls (NC) is shown. There were increases in mean diffusivity (MD) across the whole brain (A), localized decreases in fractional anisotropy (FA; B), increases in axial diffusivity (AD) across the whole brain (C), and increases in radial diffusivity (RD) across the whole brain (D), TFCE *p*<0.05. Group differences of DTI metrics are overlaid on white matter skeleton. [Color figure can be viewed at http://wileyonlinelibrary.com]

Whole‐brain analysis of the DTI data showed widespread increased MD in the pre‐HD group when compared with controls in the corpus callosum, the bilateral superior longitudinal fasciculi, the posterior limb of the internal capsules, the external capsules, the posterior thalamic radiations, the middle cerebellar peduncles, the corona radiata, the uncinate fasciculi, and the posterior cingulum (Fig 2A). This was coupled with reduced FA mainly in the corpus callosum, the superior longitudinal fasciculi, and the posterior corona radiata bilaterally (Fig 2B). Complementary to the MD findings, AD and RD both showed widespread increases in the pre‐HD group compared to the controls. We found increased AD in the corpus callosum, external capsules, right cerebellar peduncle, posterior thalamic radiations, corona radiata, and right uncinate fasciculus Fig 2C, plus increased RD in the corpus callosum, bilateral superior longitudinal fasciculi, corona radiata, left posterior limb of internal capsule, left uncinate fasciculus, and left middle cerebellar peduncle Fig 2D.

### *ROI Analysis*

In line with the whole‐brain findings, ROI analyses also showed a reduction in NDI in the splenium, body and genu of the corpus callosum, and posterior limb of the internal and external capsules and decreased ODI in the anterior limb of the internal capsules in the pre‐HD group compared with controls (Table 2).

**Table 2:** Neurite Orientation Dispersion and Density Imaging Metrics for the ROI Analysis ‐ NDI and ODI

WM ROIs	NDI, Mean ± SD		
	Pre‐HD	Control	*p*
Genu of corpus callosum	0.53 ± 0.05	0.56 ± 0.04	<0.001
Body of corpus callosum	0.58 ± 0.05	0.60 ± 0.04	0.026
Splenium of corpus callosum	0.60 ± 0.04	0.63 ± 0.03	<0.001
Posterior limb of internal capsule	0.68 ± 0.05	0.70 ± 0.04	0.023
External capsule	0.50 ± 0.03	0.51 ± 0.02	0.014

	ODI, Mean ± SD		
	Pre‐HD	Control	*p*
Anterior limb of internal capsule	0.17 ± 0.01	0.18 ± 0.01	<0.001

False discovery rate (FDR)–corrected, *p* < 0.05.

NDI = neurite density index; ODI = orientation dispersion index; Pre‐HD = premanifest Huntington's disease; ROI = region of interest; SD = standard deviation; WM = white matter.

### *NODDI Metric Correlation with Clinical Markers of Disease Progression*

**Figure 3:**
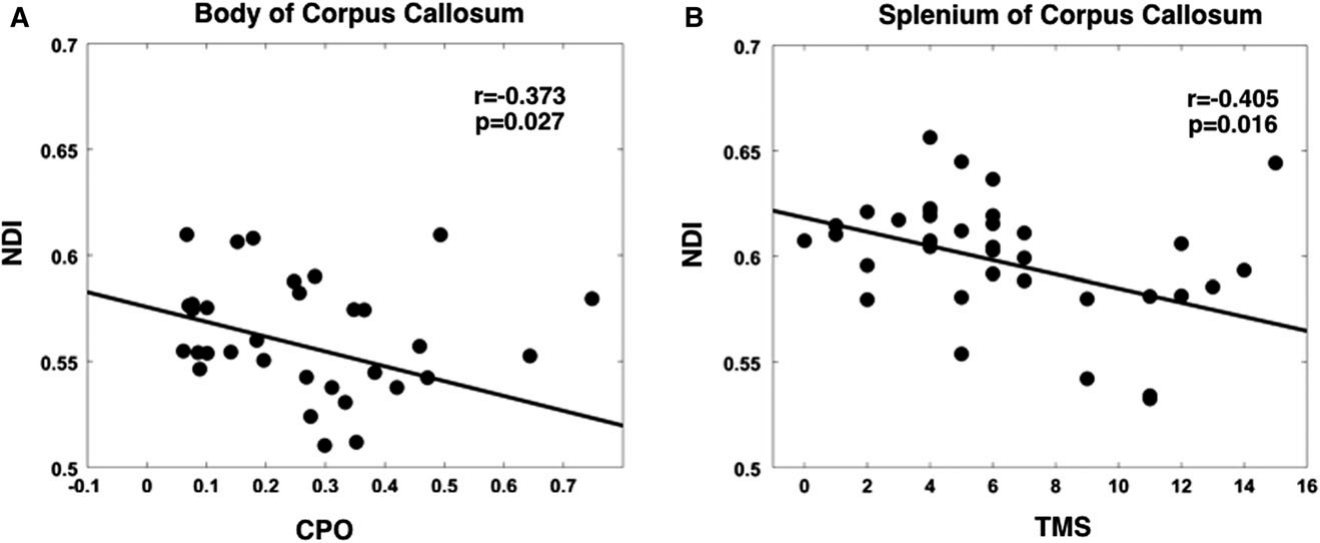
Neurite density index (NDI) correlations with clinical markers of disease progression. (A) Negative correlation between NDI in the body of the corpus callosum and cumulative probability to onset (CPO). (B) Negative correlation between NDI in the splenium of the corpus callosum and total motor score (TMS).

Both CPO and TMS were negatively correlated with NDI in the body and splenium of the corpus callosum (Fig 3); that is, reduced density was associated with increasing disease severity. ODI in the anterior limb of the internal capsules did not correlate with any clinical scores.

## Discussion

Using a pre‐HD cohort, we have provided the first detailed human in vivo characterization of white matter axonal pathology in presymptomatic neurodegeneration with multishell DWI. Using NODDI, a novel technique for analyzing multishell DWI data, we have shown a widespread reduction in axonal density (indexed by NDI) in tracts including the corpus callosum and those surrounding the basal ganglia in pre‐HD. Furthermore, axonal density reductions in callosal regions predicted clinical markers of disease progression. These findings support the view that axonal pathology is a major factor underlying white matter degeneration in pre‐HD. Interestingly, we have also shown that the coherence of axonal organization increased in those tracts surrounding the basal ganglia and in the internal and external capsules compared to controls. This suggests possible compensatory pruning of axons in white matter regions most likely affected in HD. Axonal pathology may be shared across other neurodegenerative disorders, and so our findings potentially offer new mechanistic insights into these diseases.

Widespread decreases in axonal density suggest a proliferation of impaired white matter or anatomical connections between gray matter brain regions in pre‐HD gene carriers, including the anterior and posterior limbs of the internal capsule, which connect anatomical structures within the striatum, and the striatum to both the cortex and the thalamus. These pathways are likely to be affected in HD, as the striatum is the target of the earliest neuronal loss. These NODDI findings are further supported by our independent DTI analysis, where we characterized whole‐brain changes in both FA and MD. Here, we also observed widespread MD increases in a set of tracts that overlap with those displaying axon density reductions, including the corpus callosum, and internal and external capsules. Although we cannot compare these 2 sets of findings statistically and so must proceed with caution, the NODDI and DTI analyses combined do suggest that the widespread increased diffusivity in pre‐HD can in principle be explained by reductions in the number of axons within the fibers of white matter pathways. This is highly compatible with the evidence of axonal degeneration in animal studies of pre‐HD.^12^, ^13^ It should be noted, however, that although it is biologically plausible that reduced NDI is underscored by changes in axonal density, there may also be an effect of myelin loss. Demyelinated regions may have a reduced MRI signal and be represented as reduced NDI by the NODDI model.^29^

Despite reductions in axonal density being widespread, they were coupled with localized increases in the directional coherence of axonal organization (as indexed by a decrease in ODI, ie, a refining of the complexity of the pathways) in the white matter surrounding the striatum and in the pathway linking the striatum and thalamus. These decreases in ODI suggest a process of reorganization or selective pruning of fibers within tracts linking the striatum to the cortex, potentially compensating for the reduced axonal density in the same region; this would, however, require further investigation using a more explicit model of compensation.^30^ A recent histological investigation in patients with multiple sclerosis (MS) using NODDI shows that ODI is also reduced in MS; a finding that was confirmed by histology.^29^ This study showed that in areas of intense spinal cord demyelination, orientation variability was reduced, consistent with the existing literature, and that increased organization may reflect pathologically driven reduced collateral branching or morphological alterations of individual axons. Together with this evidence, our current findings collectively support a unique and more complete picture of axonal pathology in pre‐HD.

Understanding axonal pathology in the early stages of HD could be central to developing effective therapies for use in other neurodegenerative diseases, such as Alzheimer's disease and Parkinson disease. As the underlying axonal pathology associated with the onset of neurodegeneration emerges many years before clinical symptoms, this may be the optimal point at which to administer therapeutic interventions that may delay or even prevent clinical onset. Animal models and postmortem studies are commonly used for studying disease‐related pathology, but both approaches are limited in tracking disease progression and the relationship between structure and function. Due to the low prevalence of HD in the population, this study has used a cross‐sectional approach to examine microstructural change and axonal pathology in a large group of well‐characterized participants with sufficient variability in their clinical presentation of HD symptoms. Given that only one time point has been investigated, it is possible that our findings simply represent abnormal developmental change related to the HD gene rather than neurodegenerative effects, and despite the lack of empirical evidence regarding the role of developmental abnormality, this possibility should be considered. However, we have shown that it is possible to differentiate alterations to axonal density from other potential confounding sources, including axonal spatial organization and free water contamination, in pre‐HD, which may contribute to linking ex vivo histopathological observations to modern neuroimaging findings. Further investigation in a longitudinal cohort will help to clarify the mechanistic underpinnings of these alterations.

In terms of limitations, we have used data from 2 research centers to achieve sufficient statistical power given the relatively low HD prevalence; this can give rise to potential between‐site effects from a number of sources. To minimize these potential confounding between‐site effects, we used identical acquisition protocols at both sites with the same scanner model, calibrated with a phantom, and included site as a covariate in all statistical analyses. Crossing fibers are not explicitly modeled within the NODDI model; ODI is sensitive to the presence of crossing fibers and in the case where 2 bundles of fibers cross with only 1 degenerating, reduced ODI will result.^31^ Furthermore, it should be noted that NODDI uses a relatively simple compartmental model and therefore cannot fully characterize ongoing pathology. However, it still provides a unique opportunity to investigate axonal pathology at the microscopic level, both in vivo and noninvasively. As HD is a disease characterized by movement disorders, it is important to evaluate the potential impact of motion on our findings. We therefore computed the translational motion and rotational motion based on the transformation matrix estimated for motion correction. We found that there were no significant group differences for either the translational motion (*t* = −1.733, *p* > 0.05, *df* = 81) or the rotational motion (*t* = −0.285, *p* > 0.05, *df* = 81), and as such we do not expect motion to have a significant impact on the results of our group analysis. In addition, neither translational not rotational motion correlated with the CPO measure (*p* > 0.05). However, there was an association between translational motion and NDI (*r* = −0.272, *p* < 0.05). Accordingly, we reanalyzed the correlation between TMS and NDI extracted from the splenium of the corpus callosum, including translational motion as a covariate, and found that this correlation remained significant (*r* = −0.482, *p* = 0.0039).

In the current study, we have used a novel approach to understand further the white matter microstructural changes that underlie the macrostructural alterations previously identified in premanifest HD. By using NODDI, we have examined tissue properties at the intra‐ and extracellular level and have shown widespread reduction in axonal density in pre‐HD coupled with increased organization in those areas most likely to be affected by HD pathology. Furthermore, these findings are supported both by an independent DTI analysis and the recent literature. We believe that here we present the most detailed characterization of the effects of HD pathology on white matter at the premanifest stage thus far achieved, and that the role of axonal pathology and its mechanistic underpinnings could potentially be extended to other neurodegenerative disorders.

## Author Contributions

Conception and study design: H.Z., R.I.S., A.D., S.J.T. Acquisition and analysis of data: J.Z., D.L.T., R.I.S., H.Z. Wrote the manuscript: J.Z., S.G. All authors contributed to manuscript revisions and approve the submitted article.

## Potential Conflicts of Interest

Nothing to report.
